# Monolayer MoS_2_ Bandgap Modulation by Dielectric Environments and Tunable Bandgap Transistors

**DOI:** 10.1038/srep29184

**Published:** 2016-07-05

**Authors:** Junga Ryou, Yong-Sung Kim, Santosh KC, Kyeongjae Cho

**Affiliations:** 1 Korea Research Institute of Standards and Science, Daejeon 305-340, Korea; 2 Department of Nano Science, University of Science and Technology, Daejeon 305-350, Korea; 3 Department of Materials Science and Engineering, University of Texas at Dallas, Richardson, TX 75080, USA

## Abstract

Semiconductors with a moderate bandgap have enabled modern electronic device technology, and the current scaling trends down to nanometer scale have introduced two-dimensional (2D) semiconductors. The bandgap of a semiconductor has been an intrinsic property independent of the environments and determined fundamental semiconductor device characteristics. In contrast to bulk semiconductors, we demonstrate that an atomically thin two-dimensional semiconductor has a bandgap with strong dependence on dielectric environments. Specifically, monolayer MoS_2_ bandgap is shown to change from 2.8 eV to 1.9 eV by dielectric environment. Utilizing the bandgap modulation property, a tunable bandgap transistor, which can be in general made of a two-dimensional semiconductor, is proposed.

Atomically thin two-dimensional (2D) semiconductors have attracted a great deal of attention for their superior properties in electronic devices. Monolayer (ML) molybdenum disulfide (MoS_2_) has shown high electron mobility of about 217 cm^2^ V^−1^ s^−1^ and an excessively high current on/off ratio of an order of 10^8^ in a field effect transistor (FET)^1^,^2^. However, the superior properties have been achieved only with a supporting substrate and a gate dielectric in a top gate FET structure, such as the HfO_2_/MoS_2_/SiO_2_ stack^1^,^2^. Without the top gate high-k dielectric, large reduction of the electron mobility has been reported^1^,^2^,^3^, and it has been believed to be due to the environmental dielectric screening (EDS) effect suppressing the Coulomb scattering of carriers with charged impurities in the 2D semiconductors^2^,^4^. The EDS effect has also been reported to change the defect level with the band gap and induce deep- to shallow-level transition of dopants, enhancing the carrier concentrations significantly and the electrical conductivities^5^. Furthermore, the exciton binding energies have also been reported to be affected by the EDS effect strongly in 2D semiconductors^6^,^7^.

A moderate bandgap size is a determining characteristic property of a semiconductor. Nonetheless, an accurate evaluation of the bandgap in low dimensional semiconductors has not been as simple as in conventional bulk semiconductors. It is well established that the bandgap size of MoS_2_ layers has a strong dependence on the number of layers. Furthermore, in a spatially isolated low dimensional system (e.g. freestanding 2D semiconductors), the strong unscreened Coulomb interaction (through the space outside of the 2D materials) makes the quasiparticle (QP) renormalization of electrons huge. Within the *GW* approximation, the electronic bandgap of a freestanding ML MoS_2_ has been predicted to be about 2.8 eV (refs 8, 9, 10, 11, 12). Due to the strong exciton binding (~1 eV)^8^,^9^,^10^,^11^,^12^, the optical bandgap has been obtained to be about 1.8 eV from the photoluminescence (PL) and optical absorption experiments^13^, which agree with the theoretical Bethe-Salpeter-Equation (BSE) calculations^9^,^10^,^11^,^12^. Since the exciton binding energy is large, the measured optical bandgaps are not accurate representation of semiconductor bandgap, determined by the energy difference between valence and conduction band edges. Eliminating the excitonic effect, the measurements of the electronic bandgap have given diverse results. With intercalated potassium (K) in a bulk MoS_2_, a quasi ML MoS_2_ has been fabricated from a bulk MoS_2_, and a direct bandgap of 1.86 eV at the *K* valley has been measured using angle-resolved photo-emission spectroscopy (ARPES)^14^. For a chemical vapor deposition (CVD) grown ML MoS_2_ on a Au(111) substrate, the ARPES bandgap of about 1.39 eV has been measured, which is very small^15^. In scanning tunneling spectroscopy (STS) measurements, the bandgap of a ML MoS_2_ on graphite substrate has been measured to be 2.15 eV (ref. 16) and that on a bilayer graphene to be 2.16 eV (ref. 17), which are larger than other measured values, but still significantly lower than the predicted *GW* value of 2.8 eV (refs 8, 9, 10, 11, 12). In a ML MoS_2_ phototransistor that has the Al_2_O_3_/MoS_2_/SiO_2_ stack structure, the electronic transport bandgap of the ML MoS_2_ has been measured to be 1.8 eV, in which the optically excited excitons are separated to generate electron and hole carriers by applying the source and drain bias voltages^18^. As such, the measured electronic bandgap sizes have been in a wide range (1.39–2.16 eV), and the bandgap changes have been speculated to be introduced by the EDS^10^,^15^,^17^ or carrier-induced bandgap renormalization^19^ effect. Nevertheless, the measured bandgap sizes are significantly smaller than the accurate *GW* bandgap of 2.8 eV. These findings indicate that the traditional concept of assigning a well-defined bandgap size for a specific semiconductor (e.g., 1.1 eV for Si) as a fundamental material property may not be applicable to 2D semiconductors, and that the electronic bandgap may have strong dependence on the environments. Considering the fundamental role of the bandgap size in electronic device applications, it is critical to develop a fundamental and quantitative understanding on how the environmental effects change the bandgap sizes of 2D semiconductors.

In this study, as a representative 2D semiconductor, we investigate the bandgaps of a ML MoS_2_ with various environments based on *GW* calculations, and predict a wide range of bandgap size determined by the strong effects on the ML MoS_2_ embedded in a device structure. The bandgap of ML MoS_2_ is found to change by the surrounding medium according to the dielectric constant (κ_E_) of the environment. Specifically, the *GW* bandgap changes from 2.8 eV of freestanding MoS_2_ down to about 1.9 eV for MoS_2_ in a sandwich structure between two high-k dielectrics. On the other hand, the bandgap changes down to 2.2 eV for a supported structure on ultrahigh-κ_E_ dielectrics. These *GW* bandgap changes are continuous functions of dielectric constant of the surrounding medium. Based on this finding, it is suggested that there should be transport barriers to electrons and holes in the ML MoS_2_ channel between near the metallic contacts and near the gate dielectric in a device structure, because of the different screening environments (and correspondingly different bandgap sizes) surrounding the ML MoS_2_. When the barriers are controlled by an external source, a tunable bandgap transistor can be made possible utilizing the environment-dependent property of the 2D semiconductors.

## Results and Discussion

### DFT and GW bandgaps of ML MoS_2_ with environments

**Figure 1:**
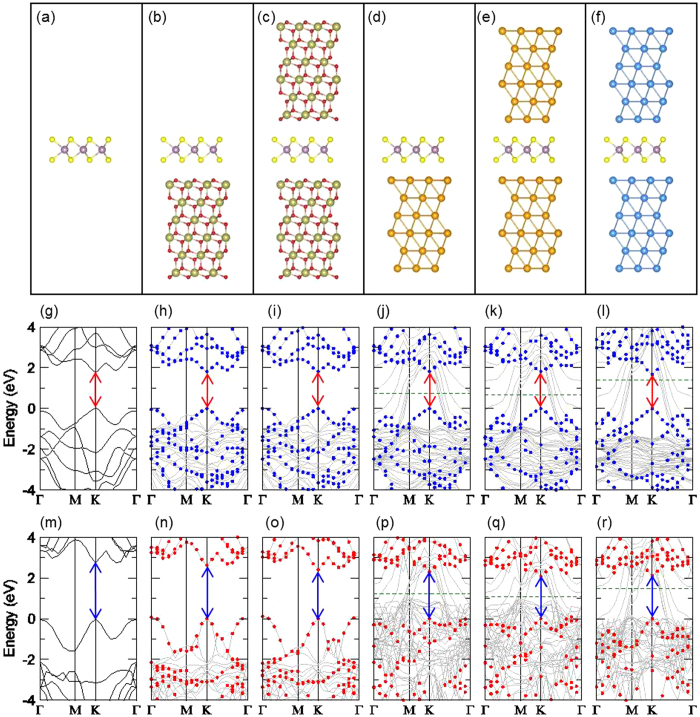
Atomic structures and electronic band structures. (**a–f**) Atomic structures of the freestanding ML MoS_2_ (**a**), ML MoS_2_ on HfO_2_ substrate (**b**), ML MoS_2_ sandwiched by HfO_2_ (**c**), ML MoS_2_ on Au metallic substrate (**d**), ML MoS_2_ sandwiched by Au (**e**), and ML MoS_2_ sandwiched by Ag (**f**). The Mo, S, Hf, O, Au, and Ag atoms are indicated by the purple, yellow, gold, red, yellow, and blue color balls, respectively. (**g–l**) Calculated LDA band structures of the freestanding ML MoS_2_ (**g**), ML MoS_2_ on HfO_2_ (**h**), ML MoS_2_ sandwiched by HfO_2_ (**i**), ML MoS_2_ on Au (**j**), ML MoS_2_ sandwiched by Au (**k**), and ML MoS_2_ sandwiched by Ag (**l**). The blue filled dots (**g–l**) indicate the projected states to the ML MoS_2_. (**m–r**) Calculated *GW* band structures of the freestanding ML MoS_2_ (**m**), ML MoS_2_ on HfO_2_ (**n**), ML MoS_2_ sandwiched by HfO_2_ (**o**), ML MoS_2_ on Au (**p**), ML MoS_2_ sandwiched by Au (**q**), and ML MoS_2_ sandwiched by Ag (**r**) are shown. The red filled dots (**m–r**) indicate the projected states to the ML MoS_2_. The arrows (**g–r**) indicate the direct bandgap at the *K* valley of the ML MoS_2_. The Fermi levels of the Au and Ag containing systems (**j–l**,**p–r**) are indicated by the green dashed lines.

First, we consider five ML MoS_2_ model systems with different dielectric environments for full DFT and *GW* calculations: (A) a freestanding ML MoS_2_ surrounded by vacuum, (B) ML MoS_2_ on a HfO_2_ substrate, (C) ML MoS_2_ sandwiched by HfO_2_, (D) ML MoS_2_ on a Au substrate, and (E) ML MoS_2_ sandwiched by Au. We construct the model atomic structures for the five systems as shown in Fig. 1a–e. The details of the structures are described in the Supplementary Materials. The calculated band structures within the local density approximation (LDA) are plotted in Fig. 1g–k. The LDA band structures indicate that the ML MoS_2_’s have a direct bandgap of 1.8 eV at the *K* valley irrespective of the environments. The electronic structure analysis shows that the orbitals of the valence band maximum (*E*_V_) and the conduction band minimum (*E*_C_) at the *K* valley are characterized as the Mo 4*d* atomic orbitals. These atomic orbitals are located in the middle of the three atomic layers of ML MoS_2_ layer, and they have negligible hybridization with the orbitals of the nearby surrounding materials that interacts with the ML MoS_2_ through the van der Waals gap. The LDA bandgap at the *K* valley is about 1.8 eV agreeing with previous DFT calculations and found to remain unchanged with different environments. For the metallic Au environments (Fig. 1j,k), the Au related states (6*s*) are found inside the bandgap of the ML MoS_2_, but they can be clearly distinguished from the ML MoS_2_ states (shown as blue dots). Within the LDA, the Au 5*d* states are found inside the valence bands of the ML MoS_2_, and the Fermi level (*E*_F_) is found to be located at *E*_V_ + 0.8 eV or *E*_C_ − 1.0 eV.

The calculated *GW* band structures of the ML MoS_2_ with the same environments are plotted in Fig. 1m–q. They also show that the ML MoS_2_ has a direct bandgap at the *K* valley irrespective of the environments, as in the case of the LDA. The direct bandgap at the *K* valley of the freestanding ML MoS_2_ (Fig. 1m) is 2.8 eV in *GW*, close to the previous calculations^8^,^9^,^10^,^11^,^12^. The *GW* bandgap of the ML MoS_2_ on HfO_2_ (Fig. 1n) is calculated to be 2.6 eV, and that of the ML MoS_2_ sandwiched by HfO_2_ (Fig. 1o) is 2.4 eV, which are smaller than that of the freestanding ML MoS_2_ (2.8 eV). Since the electronic orbital hybridization between the MoS_2_ and the nearby HfO_2_ dielectric is negligible, as shown in the LDA results (Fig. 1h,i), the main cause of the bandgap reduction in the *GW* calculations is expected to be the EDS effect on the QP bandgap renormalization. The size of the bandgap reduction is significant, up to by 0.4 eV, in the presence of HfO_2_ layers. The ML MoS_2_ on the Au metallic substrate (Fig. 1p) is found to have a direct bandgap of 2.3 eV at the *K* valley in the *GW* calculations, and the ML MoS_2_ sandwiched by the Au layers (Fig. 1q) has a *GW* bandgap of 2.1 eV. The bandgap reduction is even more significant, up to by 0.7 eV, by the metallic Au environments.

### GW bandgaps of ML MoS_2_ with effective medium

In order to investigate the primary effect of EDS on the bandgap of ML MoS_2_, we incorporate the effective environmental dielectric constant (κ_E_) into the dielectric matrix of the screened Coulomb (*W*) interaction in *GW* calculations. Details of the procedure are described in the Supplementary Materials. With this approach that includes the EDS effectively in *GW*, there are several advantages besides making it possible to study separately the EDS effect: reducing the computational costs of *GW* calculations with environments, and making it possible to include additional polarizability into the screened Coulomb (*W*) interaction. Note that the dielectric effect of liquid medium on ML MoS_2_ bandgap can be modeled by the effective dielectric medium. The quasiparticle renormalization in the *GW* approximation (Fig. 1m-r) includes only the electronic contribution of screening into the screened Coulomb (*W*) interaction. Since high-κ_E_ dielectrics such as HfO_2_ (ε_0_ ≅ 26) usually have large ionic contribution (ε_0_−ε_∝_ ≅ 21) to the dielectric screening, the renormalization of electrons in the ML MoS_2_ would be further modified by the ionic screening of the surrounding materials. However, this ionic screening effect is neglected in the *GW* calculations of the band structures shown in Fig. 1n–r. In order to include such an ionic contribution of screening explicitly, the *GW* plus the lattice polarization effect (LPE) can be applied to the calculation of the bandgap renormalization. In some ionic solids, the inclusion of the LPE has been reported to lead to a large shrinkage of the bandgap^20^,^21^,^22^. Compared to full *GW* + LPE, effective dielectric medium method provide an alternative efficient approach to include full dielectric effects on the MoS_2_ bandgap.

**Figure 2:**
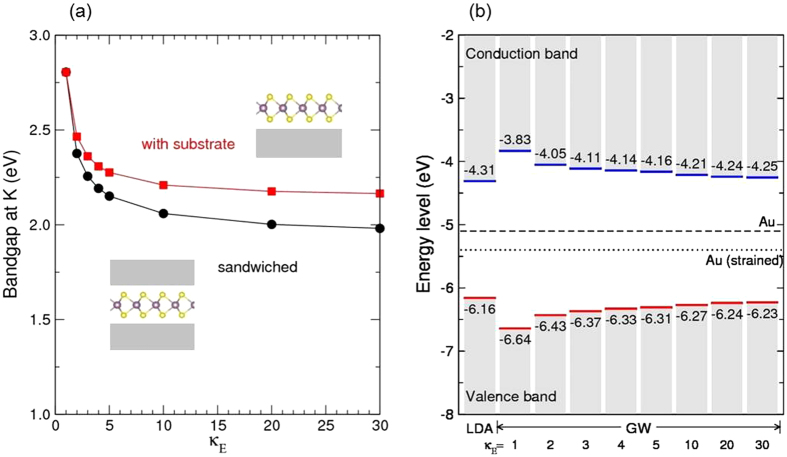
Bandgap and absolute band edge levels. (**a**) Calculated *GW* bandgaps at the *K* valley of the ML MoS_2_ on a substrate (red) and in a sandwich structure (black), as a function of the effective dielectric constant κ_E_ of the environment. (**b**) Calculated absolute *GW* band edge levels of the ML MoS_2_ sandwiched by the effective dielectric media having κ_E_. They are compared to the absolute band edge levels in LDA. The work function levels of Au (at −5.1 eV) and the strained (used in our calculations) Au (at −5.4 eV) are indicated by the dashed and dotted line, respectively.

The calculated *GW* bandgaps of ML MoS_2_ with an effective dielectric constant (κ_E_) of the environments are plotted as a function of the κ_E_ in Fig. 2a. With κ_E_ = 1, the ML MoS_2_ represents the freestanding isolated one in vacuum, and the *GW* bandgap is found to be 2.8 eV at the *K* valley. With increasing the dielectric constant, κ_E_, the *GW* bandgap is found to drop rapidly down. With the one-side dielectric, in such a case of the supported ML MoS_2_ on a substrate, the *GW* bandgap is found to reduce down to about 2.2 eV with a ultrahigh-κ_E_ dielectric (at κ_E_ = 30). With the both-side dielectric, as in a typical top-gate FET structure, the *GW* bandgap of the ML MoS_2_ is found to be smaller down to about 1.9 eV with a ultrahigh-κ_E_ dielectric of κ_E_ = 30. It is notable that the bandgap reduction is very rapid in the range 1 < κ_E_ < 5, and most of the bandgap reduction, about 80%, occurs with κ_E_ = 5. Thus, the presence of a moderate dielectric material in vicinity of a ML MoS_2_, can strongly affect the bandgap renormalization in the ML MoS_2_, even though it is not an ultrahigh-κ_E_ dielectric.

The experimentally measured bandgap of 2.15 eV in STS^16^ for the ML MoS_2_ on graphite substrate is close to the obtained asymptotic value of 2.2 eV for the one-side dielectric, and the STS bandgap of 2.16 eV for the ML MoS_2_ on a bilayer graphene substrate^17^ is also close the value. The measured ARPES bandgap of 1.86 eV for the K-intercalated MoS_2_ (ref. 14) is close to the obtained bandgap of ML MoS_2_ embedded in high-κ_E_ dielectric (1.9 eV). The measured ARPES bandgap of 1.39 eV for the ML MoS_2_ on Au substrate^15^ indicates a rather strong interaction at the interface, as shown in our previous metal-MoS_2_ interface study^18^. The measured bandgap of 1.8 eV for the ML MoS_2_ in the Al_2_O_3_/MoS_2_/SiO_2_ stacked top-gate FET^19^ is closer to the asymptotic value of 1.9 eV obtained for the both-side dielectric system. An additional reduction of the bandgap may be possible by the carrier-induced renormalization of bandgap^20^ in *n*-type ML MoS_2_ FET.

### Absolute band edge levels of ML MoS_2_ with environments

We now investigate the absolute band edge levels (relative to vacuum level) of ML MoS_2_ with including the EDS effect. Figure 2b shows the band edge levels of ML MoS_2_ in LDA and those in *GW* with various κ_E_. The *E*_V_ and *E*_C_ in LDA are found to be −6.16 and −4.31 eV, respectively, which are close to the previous LDA calculations (−5.98 and −4.29 eV)^23^. The absolute *GW* band edge levels are obtained using the bandgap center alignment scheme^23^, and the obtained *E*_V_ and *E*_C_ in *GW* with κ_E_ = 1 are −6.64 and −3.83 eV, respectively, in good agreement with the previous *GW* calculations (−6.50 and −3.74 eV, respectively)^23^. The calculated *GW* band edge levels with κ_E_ plotted in Fig. 2b show that the *E*_V_ increases up and the *E*_C_ decreases down monotonically with increasing κ_E_, approaching the LDA values of *E*_V_ and *E*_C_ with ultrahigh-κ_E_.

For the (strained) Au metal, the work function (5.4 eV) level is located at *E*_V_ + 0.8 eV or *E*_C_ − 1.1 eV with the LDA band edge levels. They agree with those obtained in our atomistic *GW* calculations (*E*_V_ + 0.8 eV or *E*_C_ − 1.0 eV) (Fig. 1j,k). In the atomistic *GW* calculations, the *E*_F_ is found to be located at *E*_V_ + 1.0 eV or *E*_C_ − 1.3 eV in the Au supported structure and at *E*_V_ + 0.9 eV or *E*_C_ − 1.2 eV in the Au sandwiched structure (Fig. 1p,q). If we use the *GW* band edge levels without EDS (κ_E_ = 1), the Au work function level is located at *E*_V_ + 1.2 eV or *E*_C_ − 1.6 eV, which is far from the atomistic *GW* calculations (*E*_V_ + 0.9 eV or *E*_C_ − 1.2 eV, as shown in Fig. 1q). When we use the *GW* band edge levels with ultrahigh-κ_E_ (says κ_E_ = 30), the Au work function level of *E*_V_ + 0.8 eV or *E*_C_ − 1.1 eV is close to the atomistic *GW* results (*E*_V_ + 0.9 eV or *E*_C_ − 1.2 eV) (Fig. 1q). Note that within the *GW*, the Au 5*d* states are found to emerge inside the bandgap of ML MoS_2_ (Fig. 1p,q), which is in contrast to the LDA results (Fig. 1j,k). Although both the LDA and *GW* results indicate that the Au work function level is located deep inside the bandgap of ML MoS_2_, and some experiments have shown that Au produces *n*-type Schottky contacts to ML MoS_2_ (refs 24,25). Au has been typically used as a *n*-type contact metal to MoS_2_ (refs 1,26,27), which may be due to the Fermi level pinning at the interface near to the *E*_C_ of MoS_2_ (refs 18,27, 28, 29).

In our calculations, the Au(111) slab is strained by +9.6% (tensile) to match in lattice to the 1 × 1 ML MoS_2_. In order to match the lattice constant of Au with the MoS_2_ within a few % of strain, a larger supercell, for example 2 × 2 Au(111) slab and √3 × √3R30° MoS_2_ with 5.0% of compressive strain, is required with the number of atoms exceeding 33, with which the *GW* calculation is computationally demanding. One effect of the tensile strain on the Au(111) slab is lowering the Fermi level with smaller band dispersions, and the work function of the strained Au(111) is 5.4 eV, while that of the unstrained Au(111) is 5.1 eV, as indicated in Fig. 2b. In both cases, the Fermi level crosses the Au(111) 6s-bands and is located inside the MoS_2_ bandgap. Both are metallic with the 6s Fermi electrons, having the infinite static dielectric constants. Since the quasi-particle bandgap dependence on the dielectric constant is very weak with the large static dielectric constant, less than 0.1 eV when κ_E_ > 10 (see Fig. 2a), the quasi-particle bandgap of the ML MoS_2_ is not expected to be significantly altered by the applied strain on the Au(111). We check another metallic slab, Ag(111), to test the quasi-particle bandgap of ML MoS_2_ with metallic screening. The Ag(111) slab is strained by 9.4% (tensile) to match with the 1 × 1 ML MoS_2_. The calculated band structures of the ML MoS_2_ with Ag(111) in the sandwich structure are shown in Fig. 1l (LDA) and Fig. 1r (*GW*). The obtained *GW* bandgap of the ML MoS_2_ is 2.11 eV with the metallic Ag(111) environment, which is nearly the same to the *GW* bandgap (2.09 eV) of the ML MoS_2_ with the metallic Au(111).

### Bandgaps of ML MoS_2_ in FET

**Figure 3:**
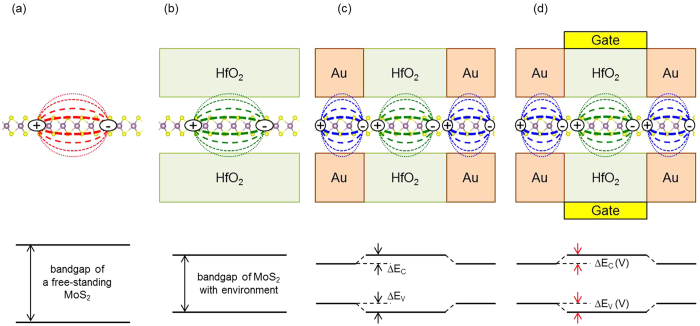
Schematic figures of screening and band diagrams. (**a**) Strong Coulomb interaction (weak screening) between electrons (red lines) in the freestanding ML MoS_2_ and the wide bandgap. (**b**) Moderate Coulomb interaction (moderate screening) between electrons (green lines) in ML MoS_2_ with both-side (HfO_2_) dielectric environments and the reduced bandgap. (**c**) Weak Coulomb interaction (strong screening) between electrons (blue lines) in ML MoS_2_ with both-side metallic (Au) environments, in conjunction with the ML MoS_2_ with both-side (HfO_2_) dielectric environments. The band offsets are indicated in the band diagram below, which act as transport barriers to electrons (Δ*E*_C_) and holes (Δ*E*_V_). (**d**) A hypothetical device structure composed of a ML MoS_2_ sandwiched by Au metallic contacts at both the ends and a dielectric material in the central region, of which the dielectric constant [κ_E_(V)] is variable with the electric field applied by the gate voltage (V). The band diagram shows the tunable transport barriers to electrons [Δ*E*_C_(V)] and holes [Δ*E*_V_(V)].

Figure 3 illustrates the effect of EDS on the bandgap of ML MoS_2_ in various environments. In an isolated freestanding ML MoS_2_, the strong Coulomb interaction (through the free space) between electrons makes the QP renormalization of electrons huge leading to a large bandgap (Fig. 3a). However, with a dielectric environment, the Coulomb interaction between electrons in the ML MoS_2_ is additionally screened, and the QP bandgap of the ML MoS_2_ is correspondingly reduced (Fig. 3b representatively for the both-side HfO_2_ dielectrics). In a typical top-gate FET device structure, a channel is located between a substrate and a gate dielectric. The channel is also connected with metallic contacts in the source and drain regions. Such typical device structure is shown in Fig. 3c with the metallic contact regions (representatively both-side Au) at both ends and the channel region with nearby dielectrics (representatively both-side HfO_2_) in the central region. For this structure, the EDS effect surrounding the ML MoS_2_ should be different for the source/drain and channel region. The different EDS strengths of metal and gate dielectric would result in different bandgaps in different regions. Under the metallic source/drain contact regions, the electronic bandgap should be smaller than in the channel region between the gate dielectric and the substrate. According to our atomistic *GW* calculations, the bandgaps in the source and drain regions with the both-side Au contacts are 2.1 eV, while the bandgap in the channel region with the both-side HfO_2_ dielectrics is 2.4 eV. The *E*_V_ and *E*_C_ offsets (Δ*E*_C_ and Δ*E*_V_) in the ML MoS_2_ channel are 0.1 and 0.2 eV, respectively. It indicates that there are an electronic transport barrier (Δ*E*_C_) of 0.2 eV from the source to the channel, and a hole transport barrier (Δ*E*_V_) of 0.1 eV, even though the ML MoS_2_ channel itself is homogeneous atomically, due to the different EDS effects (see bottom in Fig. 3c). This type of environmentally induced barriers has not been known, and can be important in low-dimensional electronic devices. Especially, they can play a role of suppressing off-state leakage current in FET by blocking the minority carrier transport.

If the κ_E_ of the gate dielectric can be controlled externally, both the electron and hole transport barriers can be controlled, as schematically illustrated in Fig. 3d. In this tunable bandgap FET, both the hole and electron transport barriers can be controlled by the gate, which is the main difference from the conventional FET that controls only the band offsets between the source/drain and the channel (and thus the transport barrier of only one type of carrier). Such tunable bandgap is highly desirable to optimize and design a novel electronic device, and bilayer graphene has been utilized to realize the tunable bandgap FET, in which the bandgap is varied by an external electric field to break the inversion symmetry of the bilayer graphene^30^. While the bilayer graphene system is highly restrictive in symmetry, the bandgap tuning by EDS can be applied generally to low dimensional semiconductors without any symmetry requirements. A challenge to realize the tunable bandgap FET by EDS is on controlling the environmental (gate) dielectric constant (κ_E_) externally. Recently, the electrically controlled dielectric materials utilizing ferroelectric properties have been suggested^31^, and such ferroelectric materials^32^,^33^,^34^,^35^ can be promising as gate dielectric materials in the EDS-based tunable bandgap FET. Polar instability at the phase transition^36^ can be also utilized to vary the dielectric constant, and distance control from the gate dielectric to the 2D semiconductor channel can be another way to control the EDS externally, which can function as an electromechanical device.

## Conclusions

Electronic bandgap of a 2D semiconductor, ML MoS_2_, depends on the nearby dielectric environments, through the screened QP renormalization of electrons in the ML MoS_2_. The bandgap tends to reduce with increasing the environmental dielectric constant. In a ML MoS_2_ FET, the vicinity of metallic contacts gives smaller bandgap than that of the gate dielectric, and there should be valence and conduction band offsets between the regions. The band offsets can play a role of barriers to electron and hole transports through the channel. Utilizing the environment-dependent property of the bandgap, a tunable bandgap FET is suggested, which operates with the bandgap controlled by an external source to control the electron and hole transport barriers.

## Methods

### Density-Functional Theory and *GW* Calculations

The mean-field density-functional theory (DFT) calculations were performed with the Quantum-Espresso code with the local density approximation (LDA)^37^. The kinetic energy cutoff for the plane-wave basis expansion of the wave-functions was 40 Ry. The 24 × 24 × 1 *k*-point sampling in the hexagonal Brillouin zone (BZ) of the 1 × 1 ML MoS_2_ was used. The *GW* calculations were performed with the Berkeley*GW* code^38^,^39^. The kinetic energy cutoff for the plane-wave basis expansion of the dielectric matrix was 6 Ry. The number of conduction bands used in the calculations of the static irreducible random phase approximation (RPA) polarizability and the Coulomb-hole self-energy was 100 for the free-standing 1 × 1 ML MoS_2_. For the 1 × 1 ML MoS_2_ with the HfO_2_ and Au environments, the number of conduction bands used was around 700. The limited number of conduction bands can affect the *GW* eigenvalues at the *M* point in the hexagonal BZ^40^, but those at the *K* point converge fast with respect to the number of conduction bands. When we used more number of conduction bands, and the *GW* bandgaps at *K* were not significantly affected. The generalized plasmon pole (GPP) approximation was used for the frequency dependence of the dielectric matrix. We applied slab truncation scheme for the Coulomb interaction to minimize the supercell interaction for the free-standing ML MoS_2_ and the ML MoS_2_ with the HfO_2_ environments.

### Model Atomic Structures

The 1 × 1 hexagonal unit cell for the ML MoS_2_ was used. The lattice constant was fixed to 3.16 Å, which is the LDA optimized value for the free-standing ML MoS_2_. With the 1 × 1 in-plane periodicity of the ML MoS_2_, the HfO_2_ was modeled by the O-terminated 1 × 1 HfO_2_(111) slab, and the Au was modeled by the 1 × 1 Au(111) slab. With these interface structures, the HfO_2_ and Au slabs are hydrostatically strained by −8.5% (compressive) and +9.6% (tensile), respectively, to match their lattice constants to that of the ML MoS_2_ (3.16 Å). The atomistic environments are only model systems that represent dielectric and metallic environments. Six Hf atomic layers were used for the HfO_2_ dielectric slab, and six Au atomic layers were used for the Au metallic slab, as shown in Fig. 1b–e in the main article. For all the interfaces, we used the interface spacing of 2.975 Å (between the atomic layers), which was chosen arbitrary as the same to the interlayer spacing between the MoS_2_ layers in bulk 2H-MoS_2_. The vacuum thickness of about 12 Å in the supercell was used. The ideal (as-cleaved) atomic structures for the HfO_2_ and Au slabs were used to see only the electronic EDS effect.

## Additional Information

**How to cite this article**: Ryou, J. *et al*. Monolayer MoS_2_ Bandgap Modulation by Dielectric Environments and Tunable Bandgap Transistors. *Sci. Rep.* **6**, 29184; doi: 10.1038/srep29184 (2016).

## Supplementary Material

Supplementary Information
