# Supplementary material for: Monolayer MoS2 Bandgap Modulation by Dielectric Environments and Tunable Bandgap Transistors
Source: Sci Rep. 2016 Jul 5;6:29184. doi: 10.1038/srep29184 (PMC4932597; doi:10.1038/srep29184)
Supplement: Supplementary Information [file srep29184-s1.pdf]

Supplementary Information

Monolayer MoS_2 Bandgap Modulation by Dielectric Environments and Tunable Bandgap Transistors

Junga Ryou[1], Yong-Sung Kim[1,2,*], Santosh KC[3] & Kyeongjae Cho[3,*]

[1]Korea Research Institute of Standards and Science, Daejeon 305-340, Korea

[2]Department of Nano Science, University of Science and Technology, Daejeon 305-350, Korea

[3]Department of Materials Science and Engineering, University of Texas at Dallas, Richardson, TX 75080, USA

* Correspondence to: Y.S.K. (email: yongsung.kim@kriss.re.kr); K.C. (email: kjcho@utdallas.edu).

Methods: *GW* Calculations with Effective Environmental Dielectric Screening

Based on classical electrostatics, the Poisson equation governing an inhomogeneous dielectric system is

$$\nabla \cdot [\varepsilon^{\mathrm{M}}(\mathbf{r})\nabla V(\mathbf{r})] = -4\pi\rho(\mathbf{r}), \tag{1}$$

where $\rho(\mathbf{r})$ is the charge density, $V(\mathbf{r})$ is the electrostatic potential, and $\varepsilon^{\mathrm{M}}(\mathbf{r})$ is the spatially varying macroscopic dielectric constant. In a periodic system with the lattice vector, **R**, the Fourier transform of Eq. (1) is

$$\sum_{\mathbf{G}'} \varepsilon^{\mathrm{M}}_{\mathbf{G}-\mathbf{G}'}\mathbf{G}\cdot\mathbf{G}' V_{\mathbf{G}'} = 4\pi\rho_{\mathbf{G}}, \tag{2}$$

where $\mathbf{G}$ and $\mathbf{G}'$ are the reciprocal lattice vectors. The Eq. 2 can be rewritten as

$$\sum_{\mathbf{G}'} \varepsilon^{\mathrm{M}}_{\mathbf{G}\mathbf{G}'} V_{\mathbf{G}'} = 4\pi\rho_{\mathbf{G}}/G^2, \tag{3}$$

$$\varepsilon^{\mathrm{M}}_{\mathbf{G}\mathbf{G}'} \equiv \varepsilon^{\mathrm{M}}_{\mathbf{G}-\mathbf{G}'}\mathbf{G}\cdot\mathbf{G}'/G^2. \tag{4}$$

Here, we define the macroscopic static dielectric matrix, $\varepsilon^{\mathrm{M}}_{\mathbf{G}\mathbf{G}'}$. On the other hand, the static dielectric matrix $\varepsilon_{\mathbf{G}\mathbf{G}'}(\mathbf{q};0)$ of a system in vacuum is related to its static polarizability $\chi_{\mathbf{G}\mathbf{G}'}(\mathbf{q};0)$ as

$$\varepsilon_{\mathbf{G}\mathbf{G}'}(\mathbf{q};0) = \delta_{\mathbf{G}\mathbf{G}'} - v(\mathbf{q}+\mathbf{G})\chi_{\mathbf{G}\mathbf{G}'}(\mathbf{q};0), \tag{5}$$

where $v(\mathbf{q}+\mathbf{G})$ is the bare Coulomb interaction. When the system is nearby a dielectric media, the additional screening by the dielectrics can be approximated by the macroscopic dielectric polarizability, $\chi^{\mathrm{M}}_{\mathbf{G}\mathbf{G}'}(\mathbf{q};0)$. $\chi^{\mathrm{M}}_{\mathbf{G}\mathbf{G}'}(\mathbf{q};0)$ is then added to the static polarizability $\chi_{\mathbf{G}\mathbf{G}'}(\mathbf{q};0)$ of the main system of interest:

$$\varepsilon_{\mathbf{G}\mathbf{G}'}(\mathbf{q};0) = \delta_{\mathbf{G}\mathbf{G}'} - v(\mathbf{q}+\mathbf{G})\{\chi_{\mathbf{G}\mathbf{G}'}(\mathbf{q};0) + \chi^{\mathrm{M}}_{\mathbf{G}\mathbf{G}'}(\mathbf{q};0)\} \tag{6}$$

$$= \varepsilon^{\mathrm{M}}_{\mathbf{G}\mathbf{G}'}(\mathbf{q};0) - v(\mathbf{q}+\mathbf{G})\chi_{\mathbf{G}\mathbf{G}'}(\mathbf{q};0). \tag{7}$$

In Eq. 7, the only difference from the static dielectric matrix of Eq. 5 is $\varepsilon^{\mathrm{M}}_{\mathbf{GG}'}(\mathbf{q};0)$ instead of $\delta_{\mathbf{GG}'}$, which represents that the vacuum is replaced by the dielectric media where the system is located. The $\varepsilon^{\mathrm{M}}_{\mathbf{GG}'}(\mathbf{q};0)$ can be obtained from Eq. 4 for the $|\mathbf{q}\rangle$ electronic state as

$$\varepsilon^{\mathrm{M}}_{\mathbf{GG}'}(\mathbf{q};0) = \varepsilon^{\mathrm{M}}_{\mathbf{q+G},\mathbf{q+G}'}$$

$$= \varepsilon^{\mathrm{M}}_{\mathbf{G-G}'}(\mathbf{q}+\mathbf{G})\cdot(\mathbf{q}+\mathbf{G}')/|\mathbf{q}+\mathbf{G}|^{2}. \quad (8)$$

The real-space macroscopic dielectric constant profiles $[\varepsilon^{\mathrm{M}}(\mathbf{r})]$ along the perpendicular direction to the ML MoS_2 are shown in Fig. S1 for the model structures of (a) the ML MoS_2 with one-side HfO_2, (b) the ML MoS_2 with both-side HfO_2, (c) the ML MoS_2 with one-side Au, and (d) the ML MoS_2 with both-side Au. The dielectric interfaces are chosen to be the top- and bottom-most atomic plane of the HfO_2 and Au slabs. At the interfaces, the dielectric profiles were smoothened with the error functions (β=0.2 Å).

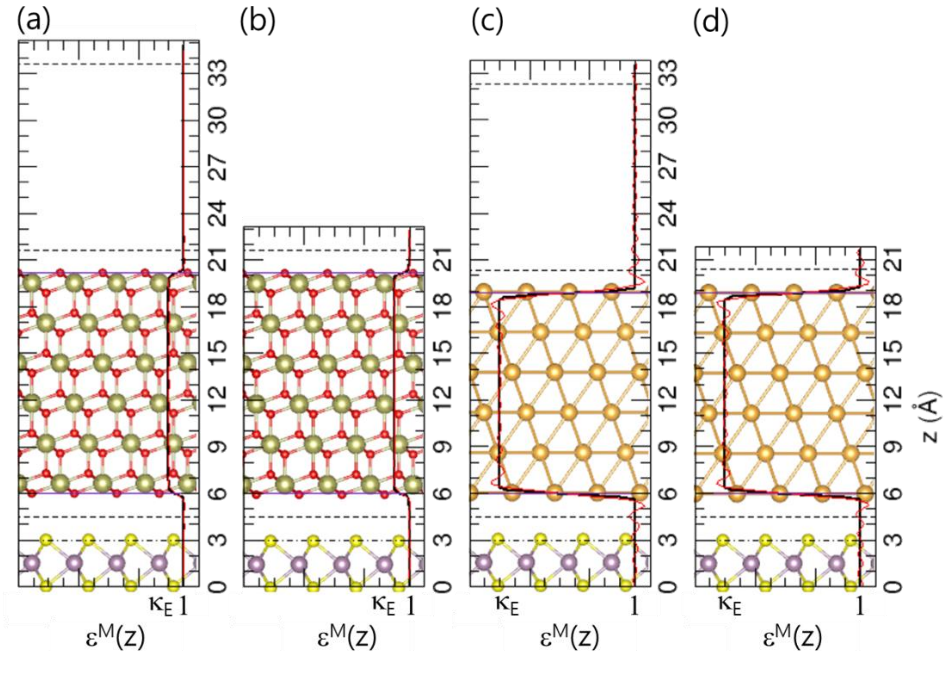

Figure S1. **Real-space environmental dielectric constant profiles.** (a-d) The dielectric constant profiles along the perpendicular direction to the ML MoS_2 for (a) the ML MoS_2 with

one-side HfO_2, (b) the ML MoS_2 with both-side HfO_2, (c) the ML MoS_2 with one-side Au, and (d) the ML MoS_2 with both-side Au.
